# Supplementary material for: Long-term risk of cardiovascular disease after assisted reproductive technology and infertility: A cohort study
Source: PLoS Med. 2026 Aug 12;23(8):e1005120. doi: 10.1371/journal.pmed.1005120 (PMC13492785; doi:10.1371/journal.pmed.1005120)
Supplement: Supplementary file 1 [file pmed.1005120.s001.docx]

Online Supplemental material

**Long-term risk of cardiovascular disease after assisted reproductive technology** **and infertility: a cohort study**

*Angelo Giosuè Mezzoiuso^1*^, Peter Henriksson^2^, Márta Radó^3,1^, Kenny A. Rodriguez-Wallberg^4^, Anna Sara Oberg^1,5^*

*1 Departments of Medical Epidemiology and Biostatistics, Karolinska Institutet, Solna, Sweden.*

*2 Department of Clinical Science, Intervention and Technology, Karolinska Institutet, Stockholm, Sweden*

*3 Centre Urbanisation Culture Société, Institut National de la Recherche Scientifique, Montréal, Quebec, Canada*

*4 Department of Oncology-Pathology, Karolinska Institutet, Stockholm, Sweden*

*5 Department of Epidemiology, Harvard T.H. Chan School of Public Health, Boston, Massachusetts, United States of America*

** angelo.giosue.mezzoiuso@ki.se*

**Table A.** Codes used to identify exposure.

| **Exposure** | **MBR** | **NPR** | | |
| --- | --- | --- | --- | --- |
|  |  | ICD-9  1987-1996 | ICD-10  since 1997 | Swedish procedural codes  since 1997 |
| Infertility | since 1982  (in years) | 628 | N97, Z350 | V23A, LCA30, DL002, DL003, DL001, DL007 |
| ART | Clinical reports since 1982.  Self-report since 1995 (yes/no) | - | Z312 |  |
| ICSI | Clinical reports since 1992.  Self-report since 1999 (yes/no) | - | Z312B |  |

**Table B**. Codes used to identify and define outcomes.

| **Outcome** | **ICD-9** | **ICD-10** |
| --- | --- | --- |
| Acute Myocardial Infarction | 410 | I21 |
| Intracranial Hemorrhage | 430-432 | I60-I62 |
| Cerebral Ischemic Conditions | 433-435 | I63, I65-I66 |
| Type 2 Diabetes Mellitus | 250 | E11 |
| Heart failure | 428 | I50 |
| Exclusion criteria for heart failure | *140-142.*  *279*  *421,424*  *745-747* | *B20,*  *I33-I39*  *Q20-Q28* |
| Aortic Aneurism/Dissection | 441 | I71 |
| Exclusion criteria for aortic aneurysm or dissection | 759W, 756W, 758G,  446, 091-097 | Q874, Q796, Q878W, Q96, M31, A51-A53 |
| Chronic Kidney Disease | 585, 403 | N18-N19, I12 |
| Exclusion criteria for chronic kidney disease | 583W, 753B, 446 | M30-M310, M311, M313, M316, M32, N02B, N11, Q6 |

**Fig A**. Directed acyclic graph of hypothesized relationships*.

* ART: Assisted reproductive technology; CVD: Cardiovascular disease; BMI: Body mass index; Origin: Country or region of origin; Genes: Genetic factors; Diseases: Pre-existing chronic diseases/comorbidities; Infertility: Infertility status; SEI: Socioeconomic Index; Age: Maternal age.

**Fig B.** Multiple imputation diagnostics: (A) MCMC convergence trace plots for the means and standard deviations of all imputed covariates; (B) density plots of observed and imputed distribution of BMI; and (C) strip plots of observed and imputed values of BMI.

**Table C.** Multiple imputation diagnostics: Observed and imputed distributions of categorical covariates.

| **Variable** | **Level** | **Observed** | **Imputed** |
| --- | --- | --- | --- |
| **SEI** | Low | 0.295 | 0.371 |
|  | Middle-low | 0.486 | 0.448 |
|  | Middle-high | 0.152 | 0.128 |
|  | High | 0.066 | 0.053 |
| **Education** | Lower Secondary | 0.135 | 0.141 |
|  | Upper Secondary | 0.555 | 0.553 |
|  | Post-secondary | 0.310 | 0.306 |
| **Smoke** | Yes | 0.848 | 0.848 |
|  | No | 0.152 | 0.152 |
| **Maternal country** | Sweden | 0.964 | 0.964 |
|  | Rest of the world | 0.036 | 0.036 |

**Table D.** Covariate distributions and standardized mean differences for the causal contrast of ART and no ART among women with primary infertility, before (population) and after (pseudo-population) inverse probability of treatment weighting.

|  | **Multiply imputed unweighted population** | | | **Multiply imputed weighted population** | | | **Multiply imputed winsorized**  **population** | | |
| --- | --- | --- | --- | --- | --- | --- | --- | --- | --- |
| **Characteristics** | **No ART** | **ART** | **SMD** | **No ART** | **ART** | **SMD** | **No ART** | **ART** | **SMD** |
| *Birth Year, M (%)* | 1996.79 (3.28) | 1997.91 (2.93) | 0.357 | 1997.14 (3.22) | 1997.43 (3.14) | 0.093 | 1997.14 (3.22) | 1997.49 (3.11) | 0.111 |
| *BMI, M (%)* | 24.33 (4.53) | 24.24 (3.92) | -0.021 | 24.31 (4.36) | 24.35 (4.35) | 0.010 | 24.31 (4.36) | 24.32 (4.23) | 0.002 |
| *Country of birth, N (%)* |  |  |  |  |  |  |  |  |  |
| Sweden | 25700 ± 1 (96.9%) | 10565 ± 0 (96.7%) |  | 25736 ± 2 (96.8%) | 10252 ± 5 (96.7%) |  | 25736 ± 2 (96.8%) | 9706 ± 5 (96.7%) |  |
| All other countries | 814 ± 1 (3.1%) | 358 ± 0 (3.3%) | 0.012 | 840 ± 1 (3.2%) | 351 ± 2 (3.3%) | 0.009 | 840 ± 1 (3.2%) | 328 ± 1 (3.3%) | 0.006 |
| *Tobacco smoking, N (%)* | 3828 ± 9 (14.4%) | 927 ± 7 (8.5%) | -0.188 | 3386 ± 7 (12.7%) | 1421 ± 10 (13.4%) | 0.020 | 3386 ± 7 (12.7%) | 1164 ± 6 (11.6%) | -0.035 |
| *Education level, N (%)* |  |  |  |  |  |  |  |  |  |
| Up to middle school | 2527 ± 3 (9.5%) | 749 ± 2 (6.9%) |  | 2341 ± 3 (8.8%) | 1027 ± 8 (9.7%) |  | 2341 ± 3 (8.8%) | 817 ± 2 (8.1%) |  |
| High School | 15062 ± 4 (56.8%) | 5551 ± 2 (50.8%) | -0.120 | 14656 ± 3 (55.1%) | 5737 ± 12 (54.1%) | -0.021 | 14656 ± 3 (55.1%) | 5428 ± 4 (54.1%) | -0.021 |
| University | 8925 ± 3 (33.7%) | 4623 ± 2 (42.3%) | 0.179 | 9578 ± 3 (36.0%) | 3839 ± 5 (36.2%) | 0.003 | 9578 ± 3 (36.0%) | 3789 ± 3 (37.8%) | 0.036 |
| *Socioeconomic index N (%)* |  |  |  |  |  |  |  |  |  |
| Low | 8554 ± 40 (32.3%) | 2670 ± 13 (24.4%) |  | 7982 ± 26 (30.0%) | 3219 ± 25 (30.4%) |  | 7982 ± 26 (30.0%) | 2892 ± 15 (28.8%) |  |
| Middle-low | 11988 ± 42 (45.2%) | 4979 ± 20 (45.6%) | 0.007 | 12045 ± 32 (45.3%) | 4774 ± 24 (45.0%) | -0.006 | 12045 ± 32 (45.3%) | 4572 ± 17 (45.6%) | 0.005 |
| Middle-high | 4161 ± 20 (15.7%) | 2202 ± 10 (20.2%) | 0.117 | 4516 ± 16 (17.0%) | 1816 ± 10 (17.1%) | 0.004 | 4516 ± 16 (17.0%) | 1776 ± 9 (17.7%) | 0.019 |
| High | 1811 ± 14 (6.8%) | 1071 ± 11 (9.8%) | 0.108 | 2032 ± 15 (7.6%) | 795 ± 7 (7.5%) | -0.006 | 2032 ± 15 (7.6%) | 794 ± 7 (7.9%) | 0.010 |
| *Age, M (%)* | 28.90 (4.64) | 32.02 (3.98) | 0.721 | 29.82 (4.68) | 30.00 (4.69) | 0.037 | 29.82 (4.68) | 30.45 (4.37) | 0.138 |
| *Asthma, N (%)* | 1464 ± 0 (5.5%) | 451 ± 0 (4.1%) | -0.065 | 1361 ± 0 (5.1%) | 534 ± 2 (5.0%) | -0.004 | 1361 ± 0 (5.1%) | 489 ± 1 (4.9%) | -0.011 |
| *IBD, N (%)* | 147 ± 0 (0.6%) | 78 ± 0 (0.7%) | 0.020 | 161 ± 0 (0.6%) | 60 ± 0 (0.6%) | -0.005 | 161 ± 0 (0.6%) | 60 ± 0 (0.6%) | -0.001 |
| *Other diseases, N (%)* | 353 ± 0 (1.3%) | 112 ± 0 (1.0%) | -0.028 | 331 ± 0 (1.2%) | 118 ± 0 (1.1%) | -0.012 | 331 ± 0 (1.2%) | 114 ± 0 (1.1%) | -0.010 |

Footnote: Continuous variables were modeled with cubic splines.

**Fig C.** Distribution of inverse probability treatment weights (mean across 20 imputed datasets) presented on a log10 scale: A) all weights; B) winsorized weights.

**Fig D.** Mirror density plot of pooled propensity scores.

**Table E.** Cumulative failure proportions at 10, 20, and 30 years, risk differences, and risk ratios for the fully-weighted comparison of ART and no ART among women with primary infertility.

|  | **Non winsorized IPW** | | | | |  | **Winsorized IPW** | | | |
| --- | --- | --- | --- | --- | --- | --- | --- | --- | --- | --- |
| **Outcome** | **Year** | **Cumulative risk (%)** | | **Risk difference**  **(95% CI)** | **Risk ratio**  **(95% CI)** |  | **Cumulative risk (%)**  **risk (%)** | | **Risk difference**  **(95% CI)** | **Risk ratio**  **(95% CI)** |
|  |  | **ART** | **No ART** |  |  |  | **ART** | **No ART** |  |  |
| Acute myocardial infarction | 10 | 0.09 | 0.06 | 0.02 (-0.13, 0.17) | 1.38 (0.24, 8.06) |  | 0.04 | 0.06 | -0.02 (-0.09, 0.04) | 0.63 (0.14, 2.75) |
|  | 20 | 0.27 | 0.42 | -0.15 (-0.33, 0.04) | 0.65 (0.34, 1.24) |  | 0.24 | 0.42 | -0.18 (-0.31, -0.05) | 0.57 (0.35, 0.91) |
|  | 30 | 0.95 | 1.24 | -0.29 (-0.63, 0.05) | 0.76 (0.54, 1.08) |  | 0.95 | 1.24 | -0.29 (-0.61, 0.04) | 0.77 (0.56, 1.06) |
| Type 2 diabetes mellitus | 10 | 0.27 | 0.36 | -0.09 (-0.22, 0.04) | 0.75 (0.48, 1.19) |  | 0.28 | 0.36 | -0.07 (-0.21, 0.06) | 0.79 (0.50, 1.25) |
|  | 20 | 1.08 | 1.36 | -0.28 (-0.54, -0.01) | 0.80 (0.63, 1.00) |  | 1.12 | 1.36 | -0.24 (-0.5, 0.03) | 0.83 (0.66, 1.03) |
|  | 30 | 3.42 | 3.67 | -0.24 (-0.88, 0.39) | 0.93 (0.78, 1.12) |  | 3.56 | 3.67 | -0.10 (-0.76, 0.55) | 0.97 (0.81, 1.17) |
| Cerebral ischemic conditions | 10 | 0.12 | 0.10 | 0.02 (-0.07, 0.11) | 1.18 (0.54, 2.59) |  | 0.12 | 0.10 | 0.02 (-0.07, 0.12) | 1.25 (0.57, 2.74) |
|  | 20 | 0.47 | 0.40 | 0.08 (-0.09, 0.25) | 1.19 (0.82, 1.74) |  | 0.49 | 0.40 | 0.09 (-0.08, 0.26) | 1.23 (0.85, 1.77) |
|  | 30 | 1.46 | 1.05 | 0.41 (0.02, 0.80) | 1.39 (1.04, 1.85) |  | 1.51 | 1.05 | 0.46 (0.07, 0.84) | 1.44 (1.09, 1.90) |
| Heart failure | 10 | 0.09 | 0.04 | 0.05 (-0.01, 0.11) | 2.26 (0.89, 5.76) |  | 0.09 | 0.04 | 0.05 (-0.01, 0.12) | 2.39 (0.94, 6.08) |
|  | 20 | 0.19 | 0.25 | -0.06 (-0.16, 0.04) | 0.76 (0.46, 1.24) |  | 0.20 | 0.25 | -0.05 (-0.15, 0.06) | 0.80 (0.49, 1.31) |
|  | 30 | 0.97 | 0.92 | 0.05 (-0.29, 0.39) | 1.06 (0.74, 1.50) |  | 1.04 | 0.92 | 0.12 (-0.24, 0.48) | 1.13 (0.80, 1.62) |
| Intracranial Hemorrhage | 10 | 0.09 | 0.09 | 0.00 (-0.07, 0.06) | 0.97 (0.46, 2.04) |  | 0.09 | 0.09 | 0.00 (-0.07, 0.07) | 1.02 (0.48, 2.16) |
|  | 20 | 0.29 | 0.31 | -0.01 (-0.16, 0.14) | 0.96 (0.58, 1.59) |  | 0.29 | 0.31 | -0.02 (-0.15, 0.12) | 0.94 (0.59, 1.50) |
|  | 30 | 0.64 | 0.64 | 0.00 (-0.24, 0.24) | 1.00 (0.69, 1.45) |  | 0.66 | 0.64 | 0.02 (-0.22, 0.26) | 1.03 (0.71, 1.48) |

**Fig E**. Kaplan–Meier failure curves of chronic kidney disease and aortic aneurysm or dissection for the fully-weighted comparison of ART and no ART among women with primary infertility, with 95% confidence intervals (shaded areas).

**Table F.** Cumulative failure proportions at 10, 20, and 30 years, risk differences, and risk ratios for the age-weighted comparison of women with and without primary infertility.

| **Outcome** | **Year** | **Cumulative risk (%)** | | **Risk difference**  **(95% CI)** | **Risk ratio**  **(95% CI)** |
| --- | --- | --- | --- | --- | --- |
|  |  | **Infertility** | **No infertility** |  |  |
| Acute myocardial infarction | 10 | 0.03 | 0.03 | 0.01 (-0.01, 0.03) | 1.29 (0.70, 2.36) |
|  | 20 | 0.27 | 0.18 | 0.09 (0.03, 0.14) | 1.51 (1.21, 1.87) |
|  | 30 | 0.93 | 0.63 | 0.29 (0.16, 0.43) | 1.47 (1.26, 1.71) |
| Type 2 diabetes mellitus | 10 | 0.34 | 0.17 | 0.17 (0.10, 0.24) | 2.01 (1.62, 2.5) |
|  | 20 | 1.29 | 0.72 | 0.57 (0.44, 0.71) | 1.80 (1.61, 2.00) |
|  | 30 | 3.40 | 2.16 | 1.24 (0.97, 1.52) | 1.58 (1.45, 1.71) |
| Cerebral ischemic conditions | 10 | 0.09 | 0.07 | 0.02 (-0.02, 0.05) | 1.23 (0.83, 1.84) |
|  | 20 | 0.40 | 0.32 | 0.08 (0.00, 0.16) | 1.25 (1.03, 1.52) |
|  | 30 | 0.98 | 0.85 | 0.13 (-0.01, 0.27) | 1.15 (1.00, 1.33) |
| Heart failure | 10 | 0.04 | 0.04 | 0.00 (-0.02, 0.02) | 1.09 (0.66, 1.80) |
|  | 20 | 0.19 | 0.16 | 0.03 (-0.02, 0.08) | 1.19 (0.91, 1.54) |
|  | 30 | 0.80 | 0.59 | 0.21 (0.07, 0.34) | 1.35 (1.14, 1.61) |
| Intracranial Hemorrhage | 10 | 0.06 | 0.07 | 0.00 (-0.03, 0.02) | 0.95 (0.64, 1.41) |
|  | 20 | 0.24 | 0.24 | 0.00 (-0.05, 0.06) | 1.01 (0.79, 1.28) |
|  | 30 | 0.55 | 0.58 | -0.03 (-0.13, 0.07) | 0.95 (0.79, 1.14) |

**Fig F.** Kaplan–Meier failure curves of cardiovascular outcomes for age-weighted comparison of women with and without primary infertility, with 95% confidence intervals (shaded areas).

**Table G.** Cumulative failure proportions at 10, 20, and 30 years, risk differences, and risk ratios for the fully-weighted comparison of women with and without primary infertility.

|  | **Non winsorized IPW** | | | | |  | **Winsorized IPW** | | | |
| --- | --- | --- | --- | --- | --- | --- | --- | --- | --- | --- |
| **Outcome** | **Year** | **Cumulative risk (%)** | | **Risk difference**  **(95% CI)** | **Risk ratio**  **(95% CI)** |  | **Cumulative risk (95% CI)**  **risk (%)** | | **Risk difference**  **(95% CI)** | **Risk ratio**  **(95% CI)** |
|  |  | **Infertility** | **No infertility** |  |  |  | **Infertility** | **No infertility** |  |  |
| Acute myocardial infarction | 10 | 0.03 | 0.03 | 0.00 (-0.02, 0.02) | 0.98 (0.53, 1.83) |  | 0.03 | 0.03 | 0.00 (-0.01, 0.02) | 1.17 (0.65, 2.11) |
|  | 20 | 0.22 | 0.18 | 0.04 (-0.01, 0.10) | 1.24 (0.96, 1.59) |  | 0.24 | 0.18 | 0.06 (0.01, 0.12) | 1.36 (1.09, 1.69) |
|  | 30 | 0.86 | 0.64 | 0.22 (0.05, 0.39) | 1.34 (1.10, 1.64) |  | 0.87 | 0.64 | 0.23 (0.10, 0.36) | 1.36 (1.16, 1.59) |
| Type 2 diabetes mellitus | 10 | 0.27 | 0.18 | 0.09 (0.03, 0.16) | 1.53 (1.18, 1.98) |  | 0.28 | 0.18 | 0.11 (0.05, 0.17) | 1.61 (1.28, 2.01) |
|  | 20 | 1.05 | 0.74 | 0.31 (0.18, 0.45) | 1.42 (1.24, 1.62) |  | 1.10 | 0.74 | 0.36 (0.24, 0.48) | 1.48 (1.32, 1.66) |
|  | 30 | 2.80 | 2.21 | 0.59 (0.31, 0.86) | 1.27 (1.15, 1.40) |  | 2.96 | 2.21 | 0.75 (0.50, 1.00) | 1.34 (1.23, 1.46) |
| Cerebral ischemic conditions | 10 | 0.09 | 0.07 | 0.02 (-0.03, 0.06) | 1.22 (0.76, 1.96) |  | 0.09 | 0.07 | 0.02 (-0.02, 0.05) | 1.21 (0.81, 1.82) |
|  | 20 | 0.41 | 0.32 | 0.09 (-0.01, 0.19) | 1.28 (0.99, 1.66) |  | 0.38 | 0.32 | 0.07 (-0.01, 0.14) | 1.20 (0.99, 1.47) |
|  | 30 | 0.98 | 0.86 | 0.12 (-0.06, 0.30) | 1.14 (0.95, 1.37) |  | 0.94 | 0.86 | 0.09 (-0.05, 0.22) | 1.10 (0.95, 1.27) |
| Heart failure | 10 | 0.04 | 0.04 | 0.00 (-0.02, 0.02) | 0.98 (0.59, 1.65) |  | 0.04 | 0.04 | 0.00 (-0.02, 0.03) | 1.13 (0.68, 1.89) |
|  | 20 | 0.19 | 0.17 | 0.02 (-0.04, 0.09) | 1.14 (0.80, 1.63) |  | 0.18 | 0.17 | 0.02 (-0.03, 0.07) | 1.11 (0.85, 1.44) |
|  | 30 | 0.76 | 0.60 | 0.16 (0.00, 0.32) | 1.26 (1.02, 1.57) |  | 0.75 | 0.60 | 0.15 (0.02, 0.28) | 1.24 (1.04, 1.49) |
| Intracranial Hemorrhage | 10 | 0.05 | 0.07 | -0.02 (-0.04, 0.00) | 0.73 (0.49, 1.10) |  | 0.06 | 0.07 | -0.01 (-0.03, 0.01) | 0.86 (0.58, 1.28) |
|  | 20 | 0.26 | 0.24 | 0.02 (-0.07, 0.10) | 1.07 (0.77, 1.49) |  | 0.23 | 0.24 | -0.01 (-0.06, 0.05) | 0.97 (0.76, 1.23) |
|  | 30 | 0.52 | 0.58 | -0.06 (-0.18, 0.05) | 0.89 (0.72, 1.10) |  | 0.52 | 0.58 | -0.06 (-0.16, 0.03) | 0.89 (0.75, 1.07) |

**Fig G.** Kaplan–Meier failure curves of chronic kidney disease and aortic aneurysm or dissection for fully-weighted comparison of women with and without primary infertility, with 95% confidence intervals (shaded areas).

**Fig H.** Kaplan–Meier failure curves of cardiovascular outcomes for fully-weighted comparison of ICSI and no ART among women with primary infertility, with 95% confidence intervals (shaded areas).
