# Supplementary material for: Long-term risk of cardiovascular disease after assisted reproductive technology and infertility: A cohort study
Source: PLoS Med. 2026 Aug 12;23(8):e1005120. doi: 10.1371/journal.pmed.1005120 (PMC13492785; doi:10.1371/journal.pmed.1005120)
Supplement: Supplementary file 2 [file pmed.1005120.s002.doc]

**Long-term risk of cardiovascular disease after assisted reproductive technology and infertility**

Angelo Giosuè Mezzoiuso^1^*, Peter Henriksson^2^, Márta Radó^3,1^, Kenny A. Rodriguez-Wallberg^4^, Anna Sara Oberg^1,5^

1 Departments of Medical Epidemiology and Biostatistics, Karolinska Institutet, Solna, Sweden.

2 Department of Clinical Science, Intervention and Technology, Karolinska Institutet, Stockholm, Sweden

3 Centre Urbanisation Culture Société, Institut National de la Recherche Scientifique, Montréal, Quebec, Canada

4 Department of Oncology-Pathology, Karolinska Institutet, Stockholm, Sweden

5 Department of Epidemiology, Harvard T.H. Chan School of Public Health, Boston, Massachusetts, United States of America

* angelo.giosue.mezzoiuso@ki.se

STROBE Checklist for cohort studies

|  | Item No | Recommendation | Location in Manuscript (Page/Section) |
| --- | --- | --- | --- |
| **Title and abstract** | 1 | (*a*) Indicate the study’s design with a commonly used term in the title or the abstract | Abstract (Methods and Findings), para 2. |
|  |  | (*b*) Provide in the abstract an informative and balanced summary of what was done and what was found | Abstract (Methods and Findings), para 2. |
| Introduction | | |  |
| Background/rationale | 2 | Explain the scientific background and rationale for the investigation being reported | Introduction, para 1-3. |
| Objectives | 3 | State specific objectives, including any prespecified hypotheses | Introduction, para 4.  Methods (Study hypothesis), para 1. |
| Methods | | |  |
| Study design | 4 | Present key elements of study design early in the paper | Methods (Data sources), para 2. |
| Setting | 5 | Describe the setting, locations, and relevant dates, including periods of recruitment, exposure, follow-up, and data collection | Methods (Data sources and Study Population) para 2-5. |
| Participants | 6 | (*a*) Give the eligibility criteria, and the sources and methods of selection of participants. Describe methods of follow-up | Methods (Study Population), par 4-5 and Figure 1. |
|  |  | (*b*) For matched studies, give matching criteria and number of exposed and unexposed | Not applicable, it was not a matched study. |
| Variables | 7 | Clearly define all outcomes, exposures, predictors, potential confounders, and effect modifiers. Give diagnostic criteria, if applicable | Methods (Exposure and outcomes), par 6.  Methods (Covariates), par 8. |
| Data sources/ measurement | 8 | For each variable of interest, give sources of data and details of methods of assessment (measurement). Describe comparability of assessment methods if there is more than one group | Methods (Data Sources), par 2-3. Methods (Exposure and outcomes), par 6. |
| Bias | 9 | Describe any efforts to address potential sources of bias | Methods (Study Population) par 4-5. Methods (Covariates) par 8.  Methods (Statistical Analysis): par 9-12 |
| Study size | 10 | Explain how the study size was arrived at | Methods (Study Population), par 4-5 and Figure 1. |
| Quantitative variables | 11 | Explain how quantitative variables were handled in the analyses. If applicable, describe which groupings were chosen and why | Methods, (Covariates), par 8. and Methods (Statistical Analysis), par 9. |
| Statistical methods | 12 | (*a*) Describe all statistical methods, including those used to control for confounding | Method (Statistical Analysis), par 9-12. |
|  |  | (*b*) Describe any methods used to examine subgroups and interactions | Methods (Statistical Analysis), par 10-12. |
|  |  | (*c*) Explain how missing data were addressed | Methods, (Statistical Analysis), par 11. |
|  |  | (*d*) If applicable, explain how loss to follow-up was addressed | Methods, (Study Population), par 4. |
|  |  | (*e*) Describe any sensitivity analyses | Methods, (Sensitivity analysis), par 13. |
| Results | | |  |
| Participants | 13 | (a) Report numbers of individuals at each stage of study—eg numbers potentially eligible, examined for eligibility, confirmed eligible, included in the study, completing follow-up, and analysed | Methods, (Study Population), par 4-5 and Figure 1. |
|  |  | (b) Give reasons for non-participation at each stage | Not applicable, population-based registry data; exclusions based on predefined eligibility criteria, not participant withdrawal or refusal. |
|  |  | (c) Consider use of a flow diagram | Figure 1 |
| Descriptive data | 14 | (a) Give characteristics of study participants (eg demographic, clinical, social) and information on exposures and potential confounders | Presented in Table 1 |
|  |  | (b) Indicate number of participants with missing data for each variable of interest | Presented in Table 1 |
|  |  | (c) Summarise follow-up time (eg, average and total amount) | Presented in Table 1 |
| Outcome data | 15 | Report numbers of outcome events or summary measures over time | Results (Primary analysis, Secondary analysis), par 5.  Tables E-G in S1 Appendix.  Fig E-H in S1 Appendix. |
| Main results | 16 | (*a*) Give unadjusted estimates and, if applicable, confounder-adjusted estimates and their precision (eg, 95% confidence interval). Make clear which confounders were adjusted for and why they were included | Results (Secondary analysis), par 4-5.  Figure 2-3.  Tables F in S1 Appendix.  Fig F in S1 Appendix. |
|  |  | (*b*) Report category boundaries when continuous variables were categorized | Table D in S1 Appendix. |
|  |  | (*c*) If relevant, consider translating estimates of relative risk into absolute risk for a meaningful time period | Not relevant to our study. |
| Other analyses | 17 | Report other analyses done—eg analyses of subgroups and interactions, and sensitivity analyses | Results (Sensitivity analysis), par 6  Fig H in S1 Appendix. |
| Discussion | | |  |
| Key results | 18 | Summarise key results with reference to study objectives | Discussion (Summary of Results), par 1. |
| Limitations | 19 | Discuss limitations of the study, taking into account sources of potential bias or imprecision. Discuss both direction and magnitude of any potential bias | Discussion (Strengths and limitations of the study), par 7. |
| Interpretation | 20 | Give a cautious overall interpretation of results considering objectives, limitations, multiplicity of analyses, results from similar studies, and other relevant evidence | Discussion, “Comparison with previous studies” par 8-9. |
| Generalisability | 21 | Discuss the generalisability (external validity) of the study results | Discussion (Strengths and limitations of the study) par 5-7. |
| Other information | | |  |
| Funding | 22 | Give the source of funding and the role of the funders for the present study and, if applicable, for the original study on which the present article is based | Source of funding |
